# Emergency contraception in university students: prevalence of use and knowledge gaps

**DOI:** 10.11606/s1518-8787.2021055003076

**Published:** 2021-11-08

**Authors:** Julia Barbian, Carolina Yumi Kubo, Caroline Soares Balaguer, Julia Klockner, Luiza Maria Venturini da Costa, Edi Franciele Ries, Valéria Maria Limberger Bayer

**Affiliations:** I Universidade Federal de Santa Maria Centro de Ciências da Saúde Departamento de Saúde Coletiva Santa MariaRS Brasil Universidade Federal de Santa Maria. Centro de Ciências da Saúde. Departamento de Saúde Coletiva. Santa Maria, RS, Brasil

**Keywords:** Students, Universities, Postcoital Contraception, Cross-sectional studies

## Abstract

**OBJECTIVE:**

Investigate prevalence of use and knowledge about emergency contraception (EC) among female university students from two higher education institutions.

**METHODS:**

Cross-sectional study with 1,740 undergraduates in the city of Santa Maria (RS), from May to October 2017. Information was collected in a semi-structured and self-administered 24-question questionnaire. The investigated variables were grouped into sociodemographic characteristics, sexual behavior, and knowledge of EC. Logistic regression was used for univariate and multivariate analysis, considering variables that presented p < 0.05. The model was adjusted using the Hosmer-Lemeshow test.

**RESULTS:**

The prevalence of EC use among undergraduates was 52.9%. However, only 11.9% of respondents received guidance on EC, especially on how to use it. Only 0.2% of the participants marked 120 hours as the maximum time of use, and 25.7% considered the EC to be abortive. EC use was associated with the age of first sexual intercourse.

**CONCLUSION:**

EC use had a high prevalence among female university students, however, several gaps in method knowledge still exists and it demonstrates the importance of discussing this issue earlier and planning actions of an informative nature.

## INTRODUCTION

Emergency contraception (EC), popularly known as the “morning-after pill”, is a hormonal contraceptive method that prevents pregnancy after sexual intercourse. Due to its postcoital nature, it is the ideal method to prevent unwanted pregnancy resulting from sexual violence, unprotected sexual intercourse, or failure in routine contraception^1^. In Brazil, the emergency method is a part of the Family Planning Standards since 1996^2^. Its inclusion was a milestone for guaranteeing women’s sexual and reproductive rights.

Unplanned pregnancy can be classified as mistimed, when the woman wanted to become pregnant later, or unwanted, when the woman did not plan to become pregnant at no time^3^. Abortion is considered an indicator of unplanned pregnancy and mainly affects younger women^4^. According to the National Abortion Survey (NAS)^5^, at the age of 40, almost one in five Brazilian women will have had an abortion, which may mean lack of knowledge about the correct use of routine and emergency contraceptive methods.

The women’s role in society changed over the years, as they acquire more autonomy, financial independence, insertion in the labor market, and prominence in academic environments. Entering universities can be the first step towards these achievements. The university is an encounter of different cultures and realities, which makes it difficult to establish an exact profile of women included in this environment. However, among them exists the common denominator of the search for knowledge and professional improvement, often associated with the need for contraceptive methods for those who want to postpone motherhood or even not have children^6^.

It would be possible to assume that university women, who have already completed elementary and high school, where subjects related to sexual education and contraceptive practices should have been addressed, would have adequate knowledge about contraceptive methods. Nevertheless, according to a study with nursing students from a university in Goiás^7^, many EC users showed doubts about the action mechanism, side effects, and medication access availability, with frequency of correct answers in the questionnaire lower than 50%. However, the need to carry out a survey with university students from all undergraduate courses is evident, and not just from the health area, since health students may have greater knowledge about EC than the general population.

Thus, and considering the importance of the conscious use of contraceptive methods, the present article investigated the knowledge and the prevalence of EC use by university women from two higher education institutions (HEIs) in order to provide subsidies for health education strategies.

## METHODS

This is a quantitative, cross-sectional observational study developed in two HEIs in the city of Santa Maria (RS), one public and the other private. The research, carried out from May to October 2017, included all female students from the two HEIs, except those under 18 years old, those who were in internship/boarding period, those absent at the time of the research, and those who refused to participate.

To calculate the sample, a total population of 11,949 students was considered: 8,790 enrolled in undergraduate courses at the public HEI, and 3,159 at the private HEI. According to Alano et al.^8^, there is an EC prevalence of 48.6% in university students. With an acceptable error of 2.5 percentage points and considering a confidence interval of 95%, it was established that 1,361 questionnaires would be applied. Approximately 10% was added to this number to avoid losses, reaching a sample of 1,500 students, proportionally divided between public HEI (1,103 students) and private HEI (396 students).

The data collection instrument was developed based on other similar studies^8,9^. The questionnaire also considered the students who had never had sexual intercourse, and those were instructed to answer only the questions about EC knowledge. A pre-test was carried out with 15 women from the public university under study to assess the readability and applicability of the questionnaire. Data from this pre-test were excluded in the research.

For data collection, a random drawing of courses offered by the universities was carried out, based on the institutions’ database. The drawing was divided into two stages: drawing of courses by HEIs’ teaching units and, later, drawing of classes (semesters) of these courses by the simple random sampling method. In both stages, the drawing was proportional to the number of students regularly enrolled in each area/course.

Variables were categorized, and the categories were maintained in the analysis based on the literature study. The knowledge about EC and the EC use were considered dependent variables; as independent variables, the student’s sociodemographic and economic profile (HEIs, course, course’s semester, age, income, religion and ethnicity), and sexual behavior (first sexual intercourse, partner and contraceptive method).

For data analysis, “initial period” was considered as the first half of the course, and “final period” to the second half (in semesters). In the regression analysis, for the final model, the lines with missing data were excluded, that is, the responses of individuals who did not answer one or more questions in the applied questionnaire.

In the data’s descriptive analysis, the frequency and measures of central tendency for the studied characteristics were determined. Data were expressed as mean, absolute and relative frequency. The association magnitude of the global level of knowledge with the EC use and the explanatory variables was estimated using odds ratio, with 95% confidence interval (95%CI), using logistic regression for univariate and multivariate analysis. Variables that obtained a value of p ≤ 0.25 in the Wald test in the univariate analysis were manually selected to start the multivariate model, using a step-by-step procedure, with backward selection. In the final model, the variables that obtained a value of p < 0.05 remained. The likelihood-ratio test was used to compare the models. The adequacy of the final models was assessed using the Hosmer-Lemeshow test. Statistical analysis was performed using the R software.

The research followed the ethical precepts established by Resolution no. 466/2012, of the Brazilian Ministry of Health, and was approved by the Research Ethics Committee of the Universidade Federal de Santa Maria (CAAE 68283317.7.0000.5346, Opinion no. 2.082.473). All students who agreed to participate in the research signed an informed consent form.

## RESULTS

The total of women who effectively participated in the study was 1,740, 73.4% enrolled in the public HEI. The age of the participants ranged between 18 and 63 years old, with an average of 21 years old. There was a higher prevalence of participants in the final stage of the course (52%), family income from 1 to 3 minimum wages (36.3%), white skin color (84.4%), and Catholic religion (49.7%). The courses with the highest number of respondents were those from the social and human sciences (26.4%) and health sciences (26%) centers. These data can be seen in Table 1.

**Table 1:** Distribution of sociodemographic and economic variables of women from two higher education institutions in Santa Maria (RS).

Characteristics	n	%
Institution (n = 1,740)		
Public HEI	1,277	73.4
Private HEI	463	26.6
Course period (n = 1,740)		
Initial	835	48.0
Final	905	52.0
Age group (n = 1,740)		
18–19	576	33.1
20–24	843	48.4
> 24	321	18.4
University centers (n = 1,740)		
Arts and language	123	7.1
Natural and exact sciences	121	7.0
Rural sciences	154	8.9
Health sciences	452	26.0
Social and human sciences	459	26.4
Education	158	9.1
Physical education and sports	54	3.1
Technology	198	11.4
Industrial technology	3	0.2
Polytechnic	18	1.0
Monthly family income (n = 1,734)		
Up to a minimum wage	141	8.1
1–3 minimum wages	628	36.2
3–6 minimum wages	507	29.2
6–9 minimum wages	242	14.0
9–12 minimum wages	120	6.9
12–15 minimum wages	50	2.9
More than 15 minimum wages	46	2.7
Skin color (n = 1,739)		
White	1,474	84.8
Black	88	5.1
Brown	158	9.1
Indigenous	3	0.2
Yellow	6	0.3
I do not know how to answer	10	0.6
Religion (n = 1,740)		
Catholic	865	49.7
Protestant	177	10.2
Spiritist	179	10.3
Jewish	1	0.1
Muslim	1	0.1
Umbanda/African	35	2.0
I do not have a religion	445	25.6
Other	37	2.1

HEI: higher education institution.

Most women had already had sexual intercourse (89.7%). The first sexual intercourse happened predominantly between 16 and 18 years old (57.2%). In addition, 76% stated that the last intercourse occurred with a partner considered to be long-term, and approximately 90% stated that they had used some contraceptive method. These data can be seen in Table 2.

**Table 2:** Distribution of variables related to sexual behavior.

Characteristics	n	%
Have you ever had sexual intercourse? (n = 1,740)		
No	179	10.3
Yes	1,561	89.7
Age of first sexual intercourse (n = 1,561)		
Less than 12 years old	5	0.3
12–15 years old	415	26.6
16–18 years old	893	57.2
More than 18 years old	248	15.9
Used contraceptive method in the last sexual intercourse (n = 1,561)^a^		
No	159	10.2
Yes	1,402	89.8
Used method (n = 1,402)^b^		
Male/female condom	863	61.6
Oral contraceptive	1,057	75.4
Intrauterine device	13	0.9
Interrupted coitus	51	3.6
Injectable contraceptive	12	0.9
Diaphragm	2	0.1
Vaginal ring	4	0.3
Calendar-based contraceptive	21	1.5
Minipill	3	0.2
Other	8	0.6
Type of partner in the last sexual intercourse (n = 1,561)		
Long-term	1,186	76.0
Short-term	375	24.0
Heard about emergency contraception (n = 1,561)		
No	7	0.4
Yes	1,554	99.6
Information source (n = 1,554)^b^		
Family member	363	23.4
Friend	975	62.7
Drugstore	195	12.5
Advertisement	379	24.4
Teacher/professor	318	20.5
Health professional	279	18.0

^a^ Except emergency contraception.

^b^ Possible to select more than one answer.

The most used contraceptive method was the oral contraceptive (OCP), referenced by 60.7% of the respondents. The proportion who claimed to have used a male/female condom in the last sexual intercourse was 49.6%. The use of condoms concomitantly with another method was observed in 43% of the responses. Coitus withdrawal and calendar-based contraceptive methods were the most used after condoms and OCP. Other methods, such as intrauterine device (IUD) and vaginal ring, were rarely mentioned.

Of the undergraduates who had had sexual intercourse, 52.9% said they had already used EC. Observing the usage by age group, the highest prevalence was between 20 and 24 years old, followed by equal to or less than 19 years old and greater than or equal to 25 years old. When asked about how many times they used EC in the last 12 months, 40.4% reported not having used it in the period. These data can be seen in Table 3.

**Table 3:** Distribution of variables related to the use of emergency contraception.

Characteristics	n	%
Used emergency contraception (n = 1,561)		
No	736	47.1
Yes	825	52.9
Used EC in the last 12 months (n = 825)		
Not once	333	40.4
One time	304	36.8
Two or three times	155	18.8
Four times or more	33	4.0
Reason for use (n = 825)		
Not using condoms	244	29.6
Condom breakage	193	23.4
Incorrect use of oral contraceptives	200	24.2
Uncertainty about the method used	221	26.8
I was not using any contraceptive method	106	12.8
Another reason	6	0.7
EC was administered how long after sexual intercourse (n = 824)^a^		
Up to 24 hours	679	82.4
24–48 hours	113	13.7
48–72 hours	18	2.2
After 72 hours	4	0.5
I do not know how to answer	10	1.2
Location where EC was acquired (n = 824)^a^		
Drugstore	823	99.9
UBSb	0	0.0
Other	1	0.1
EC was acquired with prescription (n = 824)^a^		
No	806	97.8
Yes	18	2.2
Received guidance (n = 824)a		
No	726	88.1
Yes	98	11.9
Showed an adverse reaction (n = 825)		
I had no adverse reaction	541	65.6
Nausea	91	11.0
Change in the menstrual cycle	214	25.9
Bleeding	37	4.5
Vomiting	13	1.6
Headache	54	6.6
Other	5	0.6

EC: emergency contraception.

^a^ Missing data, i.e., individual did not respond.

^b^ UBS: Healthcare Unit.

The most frequent reason for adopting the emergency method was not using a condom, followed by insecurity about the method used and incorrect use of the OCP. It is noteworthy that 96.1% of women used EC up to 48 hours after sexual intercourse, and most denied having experienced adverse reactions. The most common reactions reported were change in the menstrual cycle and nausea. Only 11.9% of respondents received guidance on EC, including information on how to use it, especially important when the dose is divided into two. An even smaller number (2.2%) purchased the drug with a prescription. Most undergraduates learned about the method by friends (62.7%) and advertisements (24.4%).

When asked about EC protection in relation to sexually transmitted infections (STIs), almost all respondents answered that it does not protect. Regarding the question about EC being abortifacient, 25.7% of undergraduates believe it is, and of these, 37.8% have already used it. Regarding what harms EC’s effectiveness, 78.2% answered “continuously usage as routine contraception”. Only 38.6% of the students marked they “take it often”, and 37% marked they “ingest before sexual intercourse”. When asked about how long after sexual intercourse EC can be used, 73.3% said they knew the maximum time and, of these, most marked 72 hours. Only 0.2% marked the maximum time of 120 hours. These data are shown in Table 4.

**Table 4:** Distribution of variables related to knowledge about emergency contraception.

Characteristics	n	%
Considers that EC protects against sexually transmitted diseases (n = 1,739)		
No	1,681	96.7
Yes	58	3.3
*Used EC (n = 58)*	*14*	*24.1*
Considers EC abortifacient (n = 1,737)		
No	1,290	74.3
Yes	447	25.7
*Used EC (n = 447)*	*169*	*37.8*
Impairs the effectiveness of EC (n = 1,740)		
Ingest it on an empty stomach	61	3.5
Ingest it with alcoholic beverage	890	51.1
Ingest it before sexual intercourse	644	37.0
Continuously usage as routine contraception	1,360	78.2
Frequently usage	672	38.6
Other	47	2.7
The time EC can be used after unprotected intercourse (n = 1,740)		
No	465	26.7
Yes	1,275	73.3
Time (n = 1,275)		
Up to 24 hours	283	22.2
*Used EC (n = 283)*	*106*	*37.4*
Up to 48 hours	312	24.5
*Used EC (n = 312)*	*158*	*50.6*
Up to 72 hours	674	52.8
*Used EC (n = 674)*	*475*	*70.4*
Up to 96 hours	2	0.2
*Used EC (n = 2)*	*2*	*100*
Up to 120 hours	3	0.2
*Used EC (n = 3)*	*1*	*33.3*
There is no time limit	1	0.1
*Used EC (n = 1)*	*0*	*0.0*

EC: emergency contraception.

Univariate analysis showed an association between EC use and age at first sexual intercourse. In the multivariate analysis, there was an association with the participant’s age and the age of first sexual intercourse. These data can be seen in Table 5.

**Table 5:** Univariate and multivariate analysis of factors associated with the emergency contraception use in a sample of higher education students, Santa Maria (RS).

Descriptors	Used EC	Univariate Analysis		Multivariate Analysis	
		
	n (%)	OR (95%CI)	p	OR (95%CI)	p
Institution					
Public HEI^d^	579 (51.9%)	1	----	----	----
Private HEI	246 (55.3%)	1.15 (0.92–1.43)	0.2245		
Age					
18–20 years old	231 (49.4%)	1		1	----
21–24 years old	433 (55.7%)	1.29 (1.02–1.62)	0.0770	1.5 (1.18–1.9)	< 0.001
> 24 years old	161 (51.1%)	1.07 (0.81–1.43)		1.4 (1.04–1.9)	0.028
Course period					
Initial	373 (51.1%)	1	0.1930	----	----
Final	452 (54.4%)	1.14 (0.94–1.39)			
Monthly income (in minimum wages)					
< 1	64 (52.0%)	1			
1–3	288 (51.2%)	0.97 (0.65–1.43)			
3–6	243 (53.2%)	1.05 (0.70–1.56)			
6–9	113 (53.6%)	1.06 (0.68–1.66)	0.5733	----	----
9–12	58 (51.8%)	0.99 (0.59–1.65)			
12–15	30 (63.8%)	1.63 (0.81–3.25)			
> 15	27 (62.8%)	1.56 (0.76–3.17)			
Skin color/race					
White	698 (52.2%)	1			
Black	39 (52.7%)	1.02 (0.64–1.63)			
Brown	80 (59.7%)	1.36 (0.94–1.95)	0.5791	----	----
Indigenous	1 (50.0%)	0.92 (0.06–14.67)			
Yellow	3 (75.0%)	2.75(0.28–26.47)			
I do not know how to answer	4 (44.4%)	0.73 (0.20–2.74)			
Religion					
Catholic	391 (49.7%)	1			
Protestant	69 (53.5%)	1.16 (0.80–1.69)			
Spiritist	98 (56.3%)	1.31 (0.94–1.82)	0.0673	-----	-----
Umbanda/African	23 (65.7%)	1.94 (0.95–3.96)			
Atheist	231 (57.0%)	1.34 (1.06–1.71)			
Other	13 (43.3%)	0.77 (0.37–1.62)			
Age of first sexual intercourse					
< 16 years old	288 (68.6%)	1		1	< 0.001
16–18 years old	452 (50.6%)	0.47 (0.37–0.60)	< 0.0001	0.46 (0.36–0.59)	< 0.001
> 18 years old	85 (34.3%)	0.24 (0.17–0.33)		0.22 (0.15–0.3)	
Used contraceptive method in the last sexual intercourse					
Yes	744 (53.1%)	1	0.6112	----	----
No	81 (50.9%)	0.92 (0.66–1.28)			
Partner					
Long-term	621 (52.4%)	1	0.4905	----	----
Short-term	204 (54.4%)	1.09 (0.86–1.37)			

EC: emergency contraception; OR: odds ratio; 95%CI: 95% confidence interval; HEI: higher education institution.

## DISCUSSION

The high prevalence of women who claimed to have used any contraceptive method in the last sexual intercourse, added to the high prevalence of EC use, suggest a desire by university students to postpone or avoid motherhood^6^. However, gaps in knowledge about the emergency method that could compromise its effectiveness were identified.

The average age of the sample can be explained by the average age of Brazilian university students, that is 25.7 years old^10^. The male population was excluded because, as showed by Pirotta and Schor^11^ in a study with students between 17 and 24 years old at an HEI in São Paulo, most of the contraceptive practice still focuses on women.

The choice of contraceptive method encompasses cultural, social, religious factors, and the type of relationship with the sexual partner^12^. It is highlighted that data analysis allows us to make a direct but incomplete reading of the subject.

The most used contraceptive method in the last intercourse was the OCP, a result different from what was observed in a study by Borges et al.^9^ with students of both sexes at a university in São Paulo, which demonstrated the male condom as the most used. Despite the increased risk of STIs, it is important to emphasize that the high prevalence of OCP use reflects greater independence in terms of sexuality and family planning, by making women responsible for their contraception^2^.

The proportion of respondents who claimed to have used a male/female condom in the last intercourse was higher compared to the Survey of Knowledge, Attitudes and Practices in the Brazilian Population aged 15-54 years old^13^, in which only 32.5% of women reported having used a condom in the last intercourse. Although EC plays a prominent role in contraception, it is worth emphasizing that condoms remain essential, as they are the only method that protects against STIs.

The proportion of women who claimed to have used EC was high when compared to a national survey conducted by the Brazilian Ministry of Health^14^ with women aged 15 to 49 years old, and the prevalence of use was 12%. The present research found results similar to those of Borges et al.^9^ (50,4%) and Alano et al.^8^ (48,6%), carried out with university students from an HEI in Santa Catarina.

The prevalent age group of EC use in this study corroborates the National Survey on Demography and Health of Women and Children^14^ and the study by Borges et al.^9^. Having the first sexual intercourse at a younger age was an independent factor associated with EC use, a result found with multivariate analysis. This association was also observed by Bastos^15^, with students from the nursing course, and by Borges et al.^9^, who found an association between EC use and having the first sexual intercourse at 17 and 18 years old or younger, respectively. In this context, special attention to women who started their sexual life earlier is recommended, providing adequate guidance for the method’s rational use. Considering these findings, it is necessary to assess the need for sexual education during elementary and high school, in order to address contraceptive practices in an early and informative way.

Demand for EC has increased over the years. A study with women who requested EC in the emergency room of a hospital in Barcelona showed that demand went from 1.26% in 1994 to 9.82% in 2002^16^. Another study, carried out in France, with women aged 15 to 24 years old, showed that 14.6% had used EC in 1999, and 31.7% in 2004^17^. This increase can be interpreted in different ways: a greater number of women are aware of the method or better family planning.

Regarding the concern that women substitute routine contraception for EC, another study showed a low prevalence of repeated use in the last 12 months and maintenance of routine methods, data suggests that one method has not been replaced by another.

The alleged reasons for using EC coincide with the criteria recommended by the Brazilian Ministry of Health^1^, such as the absence or incorrect use of contraceptive methods or condom breakage. However, the finding of EC use due to insecurity regarding routine contraception demonstrates a possible lack of information about the usual methods’ effectiveness and correct use. Similar results were found in other studies with university students ^9,15^.

Although practically all the respondents in the research have heard about EC, this does not equate to a correct understanding of its use. According to Nunes^18^, in a research with high school students in Portugal, most of the respondents knew about the existence of EC, but they lacked specific knowledge about the method. The author relates this lack of knowledge to the incomplete dissemination of information by health professionals, who are afraid to talk about EC for the fear of repeated use and irresponsible sexual behavior^18^.

Souza and Brandão^19^, by a review of the Brazilian literature, state that information about EC is generally restricted to health professionals. In the present study, this is evident when only a small portion of the respondents indicated that they learned about the method from a health professional. It is worrisome that most students know about EC from friends and advertisements, as information conveyed in this way may be incorrect.

Considering that health education is an agent of social transformation, clarification about EC is essential to ensure its rational use and reduce the number of unwanted pregnancies. Hardy et al.^20^, when investigating the facilitators and barriers to accessing EC in Brazil by discussion groups with different sectors of society, found agreement among the participants, who stated that the population has the right to know EC exists, this being a matter of citizenship. The authors also state that the barriers to accessing EC in Brazil are more linked to the individual perspectives of health professionals than to the structural resistances of society^20^. Thus, the method should be offered as another contraceptive alternative, emphasizing its emergency character.

In the present study, the pharmacy was the main place to obtain EC, a result similar to the one found by Borges et al.^9^. Even though it is a drug with a red stripe, which indicates “prescription sale”, EC is easily obtained, as evidenced by the fact that the minority of respondents in this study purchased the drug with a prescription. Low frequency of acquisition with prescription was also found by Alano et al.^8^ (2.9%) and by Borges et al.^9^(4.3%). However, the fact that prescription is not mandatory has positive perspectives. In a study^21^ carried out with discussion groups composed exclusively by women in European countries, the respondents were unanimous in emphasizing the importance of EC against unwanted pregnancies and the fact that prescriptions are not mandatory to speed up access. In Brazil, the ease of obtaining it becomes necessary due to the possible difficulty in accessing medical appointments in the public health system.

Although more effective the sooner it is administered, preferably within 72 hours^22^, the EC use can be extended to the fifth day after unprotected intercourse^23^. However, only three students answered 120 hours when asked. The fact that approximately half of the respondents answered that it can be used within 48 hours may mean that the popular name, “morning-after pill”, confuses women regarding the maximum time to administer the medication^24^.

When used frequently, the effectiveness of EC decreases due to the accumulating failure rate for each exposure^1^. In the present study, the undergraduates demonstrated knowledge about the reduction in effectiveness due to frequent use. In addition, the frequent use may be related to a previous planning of use of the emergency method. In a research by Chofakian et al.^25^, with high school students aged between 15 and 19 years old, 78.8% of the participants stated that EC should not be used before sexual intercourse, corroborating the results found in this study. The participants in this research also believe that there is a reduction in the effectiveness if alcohol is ingested. The relationship between alcohol and EC is restricted to the effects related to alcohol consumption: increased risk of vomiting and greater chance of unprotected sex^26^.

When asked if EC protects against STIs, almost all respondents correctly answered that “it does not protect”. However, 14 university students, who claimed to have used the method, responded that it protects. Comparatively, in a study by Alano et al.^8^, 85% of women knew that EC does not protect against STIs.

There is still a high prevalence of women who believe that EC is abortifacient, even among those who have already used it. Silva et al.^24^ obtained similar results in a study with undergraduate students in the health field. However, there is no scientific evidence that the drug induces abortion, as it acts by preventing fertilization^1^. The method does not interfere with implantation of a fertilized egg or in an established pregnancy^27^.

The study was developed with a population of female university students and, therefore, graduated from high school, a level of education in which topics such as human reproduction and contraception are addressed^28^. The lack of knowledge about EC among the female population outside the academic environment may be greater, given that only 12.5% of women have higher education in Brazil^29^.

For EC to be used prudently, some obstacles need to be overcome. One of the main ones is the popular name “morning-after pill”, which can be a confounding factor as to the maximum time of the medication’s administration. Although it has already fallen into disuse in health services and in academia, the term remains in popular daily life. The spread of the term “emergency contraception” is important because, in addition to eliminating the confusing expression “morning-after”, it reiterates the emergency nature of the drug^30^. For this, an educational approach on EC in schools is essential, in addition to the role of health professionals in social media, in order to disseminate the correct term and the conscientious use of EC.

The article has some limitations, including the inclusion of women with exclusive homosexual relationships and women outside the fertile period in the data analysis. However, these characteristics allows the study from assessing these women’s knowledge about the method. The study sample is representative, as it allowed access to many students from different areas, periods and institutions (public and private).

Although the results showed a high prevalence of EC use among university students, gaps in knowledge about the method were identified. By not knowing important aspects about EC, women are exposed to lack of protection. Inappropriate use of the method can impact projects of students who, as assumed from the research results, seem to not want to have children during graduation. The multivariate analysis allowed the identification of an association between having the first sexual intercourse earlier and EC use, which reinforces the importance of educational actions to address the subject early, during elementary and high school. It is also necessary to ensure that young people acquire adequate and reliable information regarding contraceptive methods, whether by social media or information projects within the universities themselves.

EC, when used rationally, is an important tool to reduce the occurrence of unplanned pregnancies and induced abortions. The findings of this research corroborate other studies, reinforcing the relevance of the subject and the need to continue conducting research in the area.
