# Supplementary material for: Emergency contraception in university students: prevalence of use and knowledge gaps
Source: Rev Saude Publica. 2021 Nov 8;55:74. doi: 10.11606/s1518-8787.2021055003076 (PMC8577535; doi:10.11606/s1518-8787.2021055003076)
Supplement: Translation

# Translation: Anticoncepção de emergência em universitárias: prevalência de uso e falhas no conhecimento

**Authors:** Julia Barbian, Carolina Yumi Kubo, Caroline Soares Balaguer, Julia Klockner, Luiza Maria Venturini da Costa, Edi Franciele Ries, Valéria Maria Limberger Bayer

**Affiliations:** I Brasil Universidade Federal de Santa Maria. Centro de Ciências da Saúde. Departamento de Saúde Coletiva. Santa Maria, RS, Brasil

**Keywords:** Estudantes, Universidades, Anticoncepção Pós-Coito, Estudos Transversais

## Abstract

**OBJETIVO:**

Investigar a prevalência de uso e o conhecimento sobre anticoncepção de emergência (AE) de mulheres universitárias de duas instituições de ensino superior.

**MÉTODOS:**

Estudo transversal com 1.740 graduandas na cidade de Santa Maria (RS), no período de maio a outubro de 2017. As informações foram coletadas por meio de questionário semiestruturado e autoaplicável de 24 questões. As variáveis investigadas foram agrupadas em características sociodemográficas, comportamento sexual e conhecimento da AE. Utilizou-se regressão logística para a análise univariada e multivariada, considerando variáveis que apresentaram p < 0,05. O modelo foi ajustado pelo teste de Hosmer-Lemeshow.

**RESULTADOS:**

A prevalência de uso da AE entre as graduandas foi de 52,9%. Contudo, apenas 11,9% das entrevistadas receberam orientação sobre a AE, principalmente no que se refere ao modo de uso. Apenas 0,2% das participantes marcou 120 horas como tempo máximo de uso, e 25,7% consideraram a AE abortiva. Houve associação entre uso da AE e idade da primeira relação sexual.

**CONCLUSÃO:**

Constatou-se alta prevalência de uso da AE entre mulheres universitárias, no entanto, ainda existem diversas lacunas no conhecimento sobre o método, o que demonstra a importância de se discutir esse assunto mais precocemente e planejar ações de caráter informativo.

## INTRODUÇÃO

A anticoncepção de emergência (AE), popularmente conhecida como “pílula do dia seguinte”, é um método contraceptivo hormonal que previne a gravidez após o intercurso sexual. Pelo caráter pós-coital, é o método ideal para prevenir gestação indesejada decorrente de violência sexual, relação sexual desprotegida ou falha na anticoncepção de rotina^1^. No Brasil, o método emergencial faz parte das Normas de Planejamento Familiar desde 1996^2^. Sua inclusão foi um marco para a garantia dos direitos sexuais e reprodutivos da mulher.

A gravidez não planejada pode ser classificada como inoportuna, quando a mulher desejava engravidar mais tarde, ou indesejada, quando a mulher não planejava engravidar em nenhum momento^3^. O aborto é considerado um indicador de gestação não planejada e acomete principalmente mulheres mais jovens^4^. Segundo a Pesquisa Nacional de Aborto (PNA)^5^, aos 40 anos, quase uma em cada cinco brasileiras terá realizado um aborto, o que pode significar desconhecimento do uso correto dos métodos contraceptivos de rotina e emergenciais.

O papel das mulheres na sociedade vem se modificando ao longo dos anos, à medida que elas adquirem mais autonomia, independência financeira, inserção no mercado de trabalho e protagonismo em ambientes acadêmicos. O ingresso em universidades pode ser o primeiro passo em direção a essas conquistas. A universidade é composta por um encontro plural de culturas e realidades diversas, o que dificulta o estabelecimento de um perfil exato de mulheres inseridas nesse ambiente. Porém, há entre elas o denominador comum da busca por conhecimento e aprimoramento profissional, muitas vezes associada à necessidade de métodos de contracepção para quem deseja postergar a maternidade ou mesmo não ter filhos^6^.

Seria possível pressupor que mulheres universitárias, que já concluíram o ensino fundamental e médio, onde deveriam ter sido abordados assuntos referentes à educação sexual e a práticas contraceptivas, teriam conhecimento adequado sobre métodos de contracepção. Contudo, segundo estudo com acadêmicas de enfermagem de uma universidade de Goiás^7^, muitas usuárias de AE mostraram ter dúvidas quanto ao mecanismo de ação, efeitos colaterais e disponibilidade de acesso do medicamento, com frequência de acertos no questionário menor que 50%. No entanto, é evidente a necessidade de realizar uma pesquisa com universitárias de todos os cursos da graduação, e não apenas da área da saúde, uma vez que estudantes da saúde podem ter conhecimento maior sobre a AE do que a população em geral.

Desse modo, e considerando a importância do uso consciente dos métodos contraceptivos, o presente estudo investigou o conhecimento e a prevalência de uso da AE por mulheres universitárias de duas instituições de ensino superior (IES) a fim de fornecer subsídios para estratégias de educação em saúde.

## MÉTODOS

Trata-se de estudo observacional quantitativo, transversal, desenvolvido em duas IES da cidade de Santa Maria (RS), uma pública e outra privada. A pesquisa, realizada no período de maio a outubro de 2017, incluiu todas as estudantes do sexo feminino das duas IES, exceto as menores de 18 anos, as que estavam em período de estágio/internato, as ausentes no momento da pesquisa e as que se negaram a participar.

Para calcular a amostra, foi considerada uma população total de 11.949 estudantes: 8.790 matriculadas nos cursos de graduação da IES pública, e 3.159 na IES privada. Conforme Alano et al.^8^, há uma prevalência de 48,6% da AE em universitários. Com erro aceitável de 2,5 pontos percentuais e considerando um intervalo de confiança de 95%, ficou estabelecido que o número de questionários a ser aplicado seria 1.361. A este número foi adicionado aproximadamente 10% para evitar perdas, chegando-se à amostra de 1.500 estudantes, dividida proporcionalmente entre IES pública (1.103 estudantes) e IES privada (396 estudantes).

O instrumento de coleta de dados foi elaborado com base em outros estudos semelhantes^8,9^. O questionário considerou também as alunas que nunca tiveram relação sexual, que eram orientadas a responder somente as questões referentes ao conhecimento sobre a AE. Realizou-se um pré-teste com 15 mulheres da universidade pública em estudo a fim de avaliar a legibilidade e a aplicabilidade do questionário. Os dados desse pré-teste não foram incluídos na pesquisa.

Para a coleta de dados, foi realizado um sorteio aleatório dos cursos oferecidos pelas universidades, com base no banco de dados das instituições. O sorteio foi dividido em duas etapas: sorteio dos cursos por unidades de ensino das IES e, posteriormente, sorteio das turmas (semestres) desses cursos pelo método de amostragem aleatória simples. Em ambas as etapas, o sorteio foi proporcional ao número de alunas regularmente matriculadas em cada área/curso.

As variáveis foram categorizadas, e as categorias foram mantidas na análise a partir do estudo da literatura. Consideraram-se variáveis dependentes o conhecimento sobre a AE e o uso da AE; como variáveis independentes, o perfil sociodemográfico e econômico das acadêmicas (IES, curso, semestre no curso, idade, renda, religião e etnia) e o comportamento sexual (primeira relação sexual, parceiro e método contraceptivo).

Para análise dos dados, considerou-se “período inicial” aquele equivalente à primeira metade do curso, e “período final” à segunda metade (em semestres). Na análise de regressão, para o modelo final, foram excluídas as linhas com dados ausentes, isto é, as respostas dos indivíduos que não responderam uma ou mais perguntas do questionário aplicado.

Na análise descritiva dos dados, determinaram-se a frequência e as medidas de tendência central para as características estudadas. Os dados foram expressos como média, frequência absoluta e relativa. A magnitude da associação do nível global de conhecimento com o uso da AE e as variáveis explicativas foi estimada por meio do *odds ratio*, com intervalo de 95% de confiança (IC95%), utilizando regressão logística para análise univariada e multivariada. As variáveis que obtiveram valor de p ≤ 0,25 no teste de Wald na análise univariada foram selecionadas manualmente para iniciar o modelo multivariado, utilizando procedimento passo a passo, com seleção para trás. No modelo final, permaneceram as variáveis que obtiveram um valor de p < 0,05. Utilizou-se o teste da razão de verossimilhança para comparar os modelos. A adequação dos modelos finais foi avaliada pelo teste de Hosmer-Lemeshow. A análise estatística foi realizada no *software* R.

A pesquisa seguiu os preceitos éticos estabelecidos pela Resolução nº 466/2012, do Ministério da Saúde, e foi aprovada pelo Comitê de Ética em Pesquisa da Universidade Federal de Santa Maria (CAAE 68283317.7.0000.5346, Parecer nº 2.082.473). Todas as estudantes que aceitaram participar da pesquisa assinaram termo de consentimento livre e esclarecido.

## RESULTADOS

Participaram efetivamente do estudo 1.740 mulheres, 73,4% matriculadas na IES pública. A idade das participantes variou entre 18 e 63 anos, com média de 21 anos. Houve maior prevalência de participantes em fase final do curso (52%), renda familiar de 1 a 3 salários-mínimos (36,3%), cor branca (84,4%) e religião católica (49,7%). Os cursos com maior número de entrevistadas foram os dos centros de ciências sociais e humanas (26,4%) e de ciências da saúde (26%). Esses dados podem ser vistos na Tabela 1.

**Tabela 1:** Distribuição das variáveis sociodemográficas e econômicas de mulheres de duas instituições de ensino superior de Santa Maria (RS).

Características	n	%
Instituição (n = 1.740)		
IES pública	1.277	73,4
IES privada	463	26,6
Período do curso (n = 1.740)		
Início	835	48,0
Final	905	52,0
Faixa etária (n = 1.740)		
18–19	576	33,1
20–24	843	48,4
> 24	321	18,4
Centros universitários (n = 1.740)		
Artes e letras	123	7,1
Ciências naturais e exatas	121	7,0
Ciências rurais	154	8,9
Ciências da saúde	452	26,0
Ciências sociais e humanas	459	26,4
Educação	158	9,1
Educação física e desportos	54	3,1
Tecnologia	198	11,4
Técnico industrial	3	0,2
Politécnico	18	1,0
Renda mensal familiar (n = 1.734)		
Até um salário-mínimo	141	8,1
1–3 salários-mínimos	628	36,2
3–6 salários-mínimos	507	29,2
6–9 salários-mínimos	242	14,0
9–12 salários-mínimos	120	6,9
12–15 salários-mínimos	50	2,9
Mais de 15 salários mínimos	46	2,7
Cor (n = 1.739)		
Branco	1.474	84,8
Negro	88	5,1
Pardo	158	9,1
Indígena	3	0,2
Amarelo	6	0,3
Não sei responder	10	0,6
Religião (n = 1.740)		
Católica	865	49,7
Protestante	177	10,2
Espírita	179	10,3
Judaica	1	0,1
Muçulmana	1	0,1
Umbanda/Africana	35	2,0
Não possuo	445	25,6
Outra	37	2,1

IES: instituição de ensino superior.

A maioria das mulheres já havia tido relações sexuais (89,7%). A primeira relação aconteceu predominantemente entre 16 e 18 anos de idade (57,2%). Além disso, 76% afirmaram que a última relação ocorreu com parceiro considerado fixo, e aproximadamente 90% afirmaram ter usado algum método contraceptivo. Esses dados podem ser vistos na Tabela 2.

**Tabela 2:** Distribuição das variáveis relativas ao comportamento sexual.

Características	n	%
Já teve relação sexual (n = 1.740)		
Não	179	10,3
Sim	1.561	89,7
Idade da primeira relação sexual (n = 1.561)		
Menos de 12 anos	5	0,3
12–15 anos	415	26,6
16–18 anos	893	57,2
Mais de 18 anos	248	15,9
Usou método contraceptivo na última relação sexual (n = 1.561)^a^		
Não	159	10,2
Sim	1.402	89,8
Método usado (n = 1.402)^b^		
Preservativo masculino/feminino	863	61,6
Anticoncepcional oral	1.057	75,4
Dispositivo intrauterino	13	0,9
Coito interrompido	51	3,6
Anticoncepcional injetável	12	0,9
Diafragma	2	0,1
Anel vaginal	4	0,3
Tabelinha	21	1,5
Minipílula	3	0,2
Outro	8	0,6
Tipo de parceiro na última relação sexual (n = 1.561)		
Fixo	1.186	76,0
Não fixo	375	24,0
Ouviu falar da anticoncepção de emergência (n = 1.561)		
Não	7	0,4
Sim	1.554	99,6
Fonte da informação (n = 1.554)^b^		
Familiar	363	23,4
Amigo	975	62,7
Farmácia	195	12,5
Propaganda	379	24,4
Professor	318	20,5
Profissional da saúde	279	18,0

^a^ Exceto anticoncepção de emergência.

^b^ Possível selecionar mais de uma resposta.

O método contraceptivo mais utilizado foi o anticoncepcional oral (ACO), referenciado por 60,7% das entrevistadas. A proporção que afirmou ter usado preservativo masculino/feminino na última relação sexual foi de 49,6%. O uso do preservativo concomitante a outro método foi observado em 43% das respostas. Coito interrompido e tabelinha foram os mais utilizados depois do preservativo e do ACO. Outros métodos, como dispositivo intrauterino (DIU) e anel vaginal, foram pouco citados.

Das graduandas que haviam tido relações sexuais, 52,9% afirmaram já ter utilizado a AE. Observando o uso por faixa etária, a maior prevalência foi entre 20 e 24 anos, seguida por iguais ou menores a 19 anos e maiores ou iguais a 25 anos. Quando questionadas sobre o número de vezes que utilizaram a AE nos últimos 12 meses, 40,4% relataram não ter utilizado no período. Esses dados podem ser visualizados na Tabela 3.

**Tabela 3:** Distribuição das variáveis relativas ao uso da anticoncepção de emergência.

Características	n	%
Utilizou a anticoncepção de emergência (n = 1.561)		
Não	736	47,1
Sim	825	52,9
Uso de AE nos últimos 12 meses (n = 825)		
Nenhuma vez	333	40,4
Uma vez	304	36,8
Duas ou três vezes	155	18,8
Quatro vezes ou mais	33	4,0
Motivo do uso (n = 825)		
Não uso do preservativo	244	29,6
Rompimento do preservativo	193	23,4
Uso incorreto do anticoncepcional oral	200	24,2
Insegurança quanto ao método utilizado	221	26,8
Não estava usando nenhum método contraceptivo	106	12,8
Outro motivo	6	0,7
Tempo, após a relação sexual, que a AE foi administrada (n = 824)^a^		
Até 24 horas	679	82,4
24–48 horas	113	13,7
48–72 horas	18	2,2
Após 72 horas	4	0,5
Não sei responder	10	1,2
Local onde a AE foi adquirida (n = 824)^a^		
Farmácia	823	99,9
UBS^b^	0	0,0
Outro	1	0,1
Adquiriu AE com receita (n = 824)^a^		
Não	806	97,8
Sim	18	2,2
Recebeu orientação (n = 824)^a^		
Não	726	88,1
Sim	98	11,9
Apresentou reação adversa (n = 825)		
Não tive reação adversa	541	65,6
Náusea	91	11,0
Alteração do ciclo menstrual	214	25,9
Sangramento	37	4,5
Vômito	13	1,6
Cefaleia	54	6,6
Outra	5	0,6

AE: anticoncepção de emergência.

^a^ Dados ausentes, ou seja, indivíduo não respondeu.

^b^ UBS: Unidade Básica de Saúde.

O motivo mais frequente para a adoção do método emergencial foi o não uso de preservativo, seguido por insegurança quanto ao método utilizado e uso incorreto do ACO. Destaca-se que 96,1% das mulheres usaram a AE até 48 horas após a relação sexual, e a maioria negou ter sentido reações adversas. As reações mais comuns relatadas foram alteração do ciclo menstrual e náusea. Apenas 11,9% das entrevistadas receberam orientação sobre a AE, incluindo informações sobre modo de uso, especialmente importantes quando a dose é dividida em duas. Um número ainda menor (2,2%) adquiriu o medicamento com receita médica. A maioria das graduandas conheceu o método por amigos (62,7%) e propagandas (24,4%).

Quando questionadas sobre a proteção da AE em relação a infecções sexualmente transmissíveis (IST), quase a totalidade das entrevistadas respondeu que não protege. Em relação à pergunta sobre a AE ser abortiva, 25,7% das graduandas consideram que sim e, dessas, 37,8% já a utilizaram. Sobre o que prejudica a eficácia da AE, 78,2% responderam “usar continuadamente como anticoncepção de rotina”. Apenas 38,6% das estudantes marcaram “tomar frequentemente”, e 37% marcaram “ingerir antes da relação sexual”. Ao serem questionadas sobre quanto tempo após o intercurso sexual a AE pode ser usada, 73,3% afirmaram saber o tempo máximo e, dessas, a maioria marcou 72 horas. Apenas 0,2% marcaram o tempo máximo de 120 horas. Esses dados constam da Tabela 4.

**Tabela 4:** Distribuição das variáveis relativas ao conhecimento acerca da anticoncepção de emergência.

Características	n	%
Considera que a AE protege de doenças sexualmente transmissíveis (n = 1.739)		
Não	1.681	96,7
Sim	58	3,3
*Usou a AE (n = 58)*	*14*	*24,1*
Considera a AE abortiva (n = 1.737)		
Não	1.290	74,3
Sim	447	25,7
*Usou a AE (n = 447)*	*169*	*37,8*
Prejudica a eficácia da AE (n = 1.740)		
Ingerir em jejum	61	3,5
Ingerir com bebida alcoólica	890	51,1
Ingerir antes da relação sexual	644	37,0
Usar continuadamente como anticoncepção de rotina	1.360	78,2
Usar frequentemente	672	38,6
Outro	47	2,7
Tempo após o intercurso desprotegido que a AE pode ser usada (n = 1.740)		
Não	465	26,7
Sim	1.275	73,3
Tempo (n = 1.275)		
Até 24 horas	283	22,2
*Usou a AE (n = 283)*	*106*	*37,4*
Até 48 horas	312	24,5
*Usou a AE (n = 312)*	*158*	*50,6*
Até 72 horas	674	52,8
*Usou a AE (n = 674)*	*475*	*70,4*
Até 96 horas	2	0,2
*Usou a AE (n = 2)*	*2*	*100*
Até 120 horas	3	0,2
*Usou a AE (n = 3)*	*1*	*33,3*
Não existe tempo-limite	1	0,1
*Usou a AE (n = 1)*	*0*	*0,0*

AE: anticoncepção de emergência.

A análise univariada demonstrou associação entre uso da AE e idade da primeira relação sexual. Na análise multivariada, verificou-se associação com a idade da participante e a idade da primeira relação sexual. Esses dados podem ser vistos na Tabela 5.

**Tabela 5:** Análise univariada e multivariada dos fatores associados com o uso da anticoncepção de emergência na amostra de estudantes de ensino superior, Santa Maria (RS).

Descritores	Já utilizou AE	Análise Univariada		Análise Multivariada	
		
	n (%)	OR (IC95%)	p	OR (IC95%)	p
Instituição					
IES^d^ pública	579 (51,9%)	1	----	----	----
IES privada	246 (55,3%)	1,15 (0,92–1,43)	0,2245		
Idade					
18–20 anos	231 (49,4%)	1		1	----
21–24 anos	433 (55,7%)	1,29 (1,02–1,62)	0,0770	1,5 (1,18–1,9)	< 0,001
> 24 anos	161 (51,1%)	1,07 (0,81–1,43)		1,4 (1,04–1,9)	0,028
Período do curso					
Inicial	373 (51,1%)	1	0,1930	----	----
Final	452 (54,4%)	1,14 (0,94–1,39)			
Renda mensal (em salários-mínimos)					
< 1	64 (52,0%)	1			
1–3	288 (51,2%)	0,97 (0,65–1,43)			
3–6	243 (53,2%)	1,05 (0,70–1,56)			
6–9	113 (53,6%)	1,06 (0,68–1,66)	0,5733	----	----
9–12	58 (51,8%)	0,99 (0,59–1,65)			
12–15	30 (63,8%)	1,63 (0,81–3,25)			
> 15	27 (62,8%)	1,56 (0,76–3,17)			
Cor/raça					
Branco	698 (52,2%)	1			
Negro	39 (52,7%)	1,02 (0,64–1,63)			
Pardo	80 (59,7%)	1,36 (0,94–1,95)	0,5791	----	----
Indígena	1 (50,0%)	0,92 (0,06–14,67)			
Amarelo	3 (75,0%)	2,75(0,28–26,47)			
Não sei responder	4 (44,4%)	0,73 (0,20–2,74)			
Religião					
Católica	391 (49,7%)	1			
Protestante	69 (53,5%)	1,16 (0,80–1,69)			
Espírita	98 (56,3%)	1,31 (0,94–1,82)	0,0673	-----	-----
Umbanda/Africana	23 (65,7%)	1,94 (0,95–3,96)			
Ateu	231 (57,0%)	1,34 (1,06–1,71)			
Outra	13 (43,3%)	0,77 (0,37–1,62)			
Idade da primeira relação sexual					
< 16 anos	288 (68,6%)	1		1	< 0,001
16–18 anos	452 (50,6%)	0,47 (0,37–0,60)	< 0,0001	0,46 (0,36–0,59)	< 0,001
> 18 anos	85 (34,3%)	0,24 (0,17–0,33)		0,22 (0,15–0,3)	
Usou método contraceptivo na última relação sexual					
Sim	744 (53,1%)	1	0,6112	----	----
Não	81 (50,9%)	0,92 (0,66–1,28)			
Parceiro					
Fixo	621 (52,4%)	1	0,4905	----	----
Não fixo	204 (54,4%)	1,09 (0,86–1,37)			

AE: anticoncepção de emergência; OR: *odds ratio*; IC95%: intervalo de confiança de 95%; IES: instituição de ensino superior.

## DISCUSSÃO

A elevada prevalência de mulheres que afirmaram ter usado algum método contraceptivo na última relação sexual, somada à alta prevalência de uso da AE, sugerem um desejo das universitárias de adiar ou evitar a maternidade^6^. No entanto, foram identificadas lacunas no conhecimento sobre o método emergencial que podem comprometer sua eficácia.

A média de idade da amostra pode ser explicada pela média de idade dos universitários brasileiros, que é de 25,7 anos^10^. A população masculina foi excluída, pois, como demonstrou estudo de Pirotta e Schor^11^ com estudantes entre 17 e 24 anos de uma IES de São Paulo, a maior parte da prática contraceptiva ainda incide sobre a mulher.

A escolha do método contraceptivo engloba fatores culturais, sociais, religiosos e o tipo de relação com o parceiro sexual^12^. Tendo em vista essa complexidade, destaca-se que a análise dos dados nos permite fazer uma leitura direta, porém incompleta do assunto.

O método contraceptivo mais utilizado na última relação foi o ACO, resultado diferente do observado em estudo de Borges et al.^9^ com alunos de ambos os sexos de uma universidade paulista, que demonstrou que o preservativo masculino foi o mais utilizado. Apesar do risco aumentado de IST, é importante ressaltar que a alta prevalência de uso do ACO reflete maior independência quanto à sexualidade e ao planejamento familiar, ao tornar a mulher responsável pela sua contracepção^2^.

A proporção de entrevistadas que afirmou ter utilizado o preservativo masculino/feminino na última relação foi maior em comparação com a Pesquisa de Conhecimentos, Atitudes e Práticas na População Brasileira de 15-54 anos^13^, na qual apenas 32,5% das mulheres relataram ter usado preservativo na última relação. Embora a AE tenha um papel de destaque na contracepção, cabe ressaltar que o preservativo permanece essencial, por ser o único método que protege contra IST.

A proporção de mulheres que alegou ter utilizado a AE foi elevada quando comparada a pesquisa nacional realizada pelo Ministério da Saúde^14^ com mulheres de 15 a 49 anos, na qual a prevalência de uso foi de 12%. A presente pesquisa encontrou resultados semelhantes aos de Borges et al.^9^ (50,4%), e de Alano et al.^8^ (48,6%), realizado com universitárias de uma IES de Santa Catarina.

A faixa etária prevalente de uso da AE neste estudo corrobora a Pesquisa Nacional de Demografia e Saúde da Criança e da Mulher^14^ e o estudo de Borges et al.^9^. Ter a primeira relação sexual mais jovem foi fator independente associado ao uso da AE, resultado encontrado por meio de análise multivariada. Essa associação também foi observada por Bastos^15^, com alunas do curso de enfermagem, e por Borges et al.^9^, que encontraram associação entre uso da AE e ter a primeira relação sexual aos 17 e 18 anos ou menos, respectivamente. Nesse contexto, recomenda-se atenção especial às mulheres que iniciaram a vida sexual mais cedo, fornecendo orientações adequadas ao uso racional do método. Considerando esses achados, é preciso avaliar a necessidade de educação sexual durante os ensinos fundamental e médio, a fim de abordar as práticas contraceptivas de forma precoce e elucidativa.

A procura pela AE tem aumentado com o passar dos anos. Estudo com mulheres que solicitaram AE na emergência de um hospital em Barcelona mostrou que a procura foi de 1,26% em 1994 para 9,82% em 2002^16^. Outro estudo, realizado na França, com mulheres de 15 a 24 anos, demonstrou que 14,6% haviam utilizado a AE em 1999, e 31,7% em 2004^17^. Tal aumento pode ser interpretado de diferentes formas: um número maior de mulheres está conhecendo o método ou há um melhor planejamento familiar.

Em relação ao receio de que as mulheres substituam a contracepção de rotina pela AE, outro estudo demonstrou baixa prevalência de uso repetido nos últimos 12 meses e manutenção dos métodos de rotina, dados que sugerem que um método não tem sido substituído por outro.

Os motivos alegados para utilizar a AE coincidem com os critérios recomendados pelo Ministério da Saúde^1^, como ausência ou uso incorreto de método contraceptivo ou rompimento do preservativo. No entanto, o achado de uso da AE por insegurança quanto à contracepção de rotina demonstra possível carência de informações sobre a eficácia e a correta utilização dos métodos usuais. Resultados semelhantes foram encontrados em outros estudos com universitários^9,15^.

Apesar de praticamente todas as entrevistadas da pesquisa já terem ouvido falar da AE, isso não equivale à correta compreensão de seu uso. Segundo Nunes^18^, em uma pesquisa com alunas de ensino médio em Portugal, a maioria das entrevistadas sabia da existência da AE, mas careciam de conhecimentos específicos sobre o método. A autora relaciona esse desconhecimento com a disseminação incompleta de informações por profissionais da saúde, que têm receio de falar sobre a AE por temerem o uso repetido e comportamentos sexuais irresponsáveis^18^.

Souza e Brandão^19^, mediante revisão da literatura brasileira, afirmam que as informações sobre a AE geralmente ficam restritas aos profissionais da saúde. No presente estudo, isso fica evidente quando apenas uma pequena parcela das entrevistadas assinala que soube do método por um profissional da saúde. É preocupante que a maioria das estudantes conheça a AE por amigos e propagandas, visto que as informações transmitidas desse modo podem estar incorretas.

Considerando que a educação em saúde é um agente de transformação social, o esclarecimento acerca da AE é fundamental para garantir seu uso racional e diminuir o número de gestações indesejadas. Hardy et al.^20^, ao investigarem os facilitadores e as barreiras de acesso à AE no Brasil por meio de grupos de discussão com diversos setores da sociedade, encontraram concordância entre os participantes, que afirmaram que a população tem direito de saber que a AE existe, sendo esta uma questão de cidadania. As autoras afirmam também que as barreiras para acessar a AE no Brasil estão mais ligadas às perspectivas individuais dos profissionais de saúde do que às resistências estruturais da sociedade^20^. Assim, o método deveria ser oferecido como mais uma alternativa contraceptiva, enfatizando seu caráter emergencial.

No presente estudo, a farmácia foi o principal local de obtenção da AE, resultado similar ao encontrado por Borges et al.^9^. Mesmo sendo um medicamento com tarja vermelha, indicando “venda sob prescrição médica”, a AE é facilmente obtida, como comprova o fato de que a minoria das entrevistadas deste estudo comprou o medicamento com receita. Baixa frequência de aquisição com receituário também foi encontrada por Alano et al.^8^ (2,9%) e por Borges et al.^9^(4,3%). No entanto, a não obrigatoriedade da prescrição tem perspectivas positivas. Em estudo^21^realizado com grupos de discussão compostos exclusivamente por mulheres em países europeus, as entrevistadas foram unânimes em enfatizar a importância da AE contra gravidez indesejada e da não obrigatoriedade da receita para agilizar o acesso. No Brasil, a facilidade de obtenção se torna necessária devido à possível dificuldade de acesso a consultas médicas no sistema de saúde público.

Apesar de mais efetiva quanto antes for administrada, preferencialmente em até 72 horas^22^, o uso da AE pode ser ampliado até o quinto dia após o intercurso desprotegido^23^. No entanto, apenas três estudantes responderam 120 horas quando questionadas. O fato de que aproximadamente metade das entrevistadas respondeu que o uso pode ser feito em até 48 horas pode significar que o nome popular, “pílula do dia seguinte”, confunde as mulheres quanto ao tempo máximo de administração da medicação^24^.

Quando utilizada frequentemente, a eficácia da AE diminui devido ao acúmulo da taxa de falha por cada exposição^1^. No presente estudo, as acadêmicas demonstraram conhecimento sobre a redução da eficácia por uso frequente. Acrescenta-se que o uso frequente pode estar relacionado a um planejamento prévio de uso do método emergencial. Em pesquisa de Chofakian et al.^25^, com estudantes do ensino médio com entre 15 e 19 anos de idade, 78,8% dos participantes afirmaram que não se deve usar a AE antes da relação sexual, corroborando o resultado encontrado neste estudo. As participantes da presente pesquisa também acreditam haver redução da eficácia com ingestão de bebida alcoólica. A relação entre álcool e a AE se restringe aos efeitos relacionados ao consumo do álcool: risco aumentado de vômitos e maior chance de relações sexuais desprotegidas^26^.

Quando perguntadas se a AE protege contra IST, quase a totalidade das entrevistadas respondeu corretamente que “não protege”. Todavia, 14 universitárias, que afirmaram já ter utilizado o método, responderam que protege. Comparativamente, em estudo de Alano et al.^8^, 85% das mulheres sabiam que a AE não protege contra IST.

Ainda há alta prevalência de mulheres que acreditam que a AE é abortiva, mesmo entre aquelas que já a utilizaram. Silva et al.^24^ obtiveram resultados semelhantes em estudo com graduandos da área da saúde. No entanto, não existe evidência científica de que o fármaco induz ao abortamento, pois age impedindo a fecundação^1^. O método não interfere na implantação de um óvulo fertilizado ou numa gravidez estabelecida^27^.

Destaca-se que o estudo foi desenvolvido com uma população de mulheres universitárias e, portanto, formadas no ensino médio, nível de ensino em que tópicos como reprodução humana e contracepção são abordados^28^. O desconhecimento acerca da AE entre a população feminina fora do ambiente acadêmico pode ser maior, dado que apenas 12,5% das mulheres têm ensino superior no Brasil^29^.

Para que a AE seja usada de forma prudente, é necessário superar alguns obstáculos. Um dos principais é o nome popular “pílula do dia seguinte”, que pode ser fator de confusão quanto ao tempo máximo de administração da medicação. Embora já tenha caído em desuso nos serviços de saúde e no meio acadêmico, o termo permanece no cotidiano popular. A propagação do termo “anticoncepção de emergência” é importante pois, além de eliminar a expressão confundidora “dia seguinte”, reitera o caráter emergencial do fármaco^30^. Para isso, é essencial uma abordagem educativa sobre a AE nas escolas, além da atuação de profissionais da saúde em mídias sociais, com o fim de disseminar o termo correto e o uso consciente da AE.

O trabalho tem algumas limitações, dentre elas a inclusão de mulheres com relações homossexuais exclusivas e mulheres fora do período fértil na análise dos dados. No entanto, essas características não impedem que o estudo afira o conhecimento dessas mulheres sobre o método. Ressalta-se que a amostra do estudo é representativa, pois possibilitou o acesso a um grande número de estudantes de diferentes áreas, períodos e instituições (pública e privada).

Embora os resultados tenham evidenciado alta prevalência de uso da AE entre universitárias, foram identificadas lacunas no conhecimento sobre o método. Ao desconhecerem aspectos importantes sobre a AE, as mulheres são expostas à desproteção. O uso inadequado do método pode impactar projetos de estudantes que, como se presume dos resultados da pesquisa, parecem não desejar ter filhos durante a graduação. A análise multivariada permitiu identificar associação entre ter a primeira relação sexual mais cedo e uso da AE, o que reforça a importância de ações de caráter educativo para abordar o assunto de forma precoce, durante o ensino fundamental e médio. Também é necessário garantir que jovens adquiram informações adequadas e confiáveis em relação aos métodos contraceptivos, seja por meio de mídias sociais ou de projetos informativos dentro das próprias universidades.

A AE, quando usada de forma racional, é uma importante ferramenta para reduzir a ocorrência de gestações não planejadas e abortamentos provocados. Os achados da presente pesquisa corroboram outros estudos, reforçando a relevância do assunto e a necessidade de continuar realizando pesquisas na área.
